# Supplementary material for: Do Bacterial “Virulence Factors” Always Increase Virulence? A Meta-Analysis of Pyoverdine Production in Pseudomonas aeruginosa As a Test Case
Source: Front Microbiol. 2016 Dec 12;7:1952. doi: 10.3389/fmicb.2016.01952 (PMC5149528; doi:10.3389/fmicb.2016.01952)
Supplement: Supplementary file 5 [file Image1.PDF]

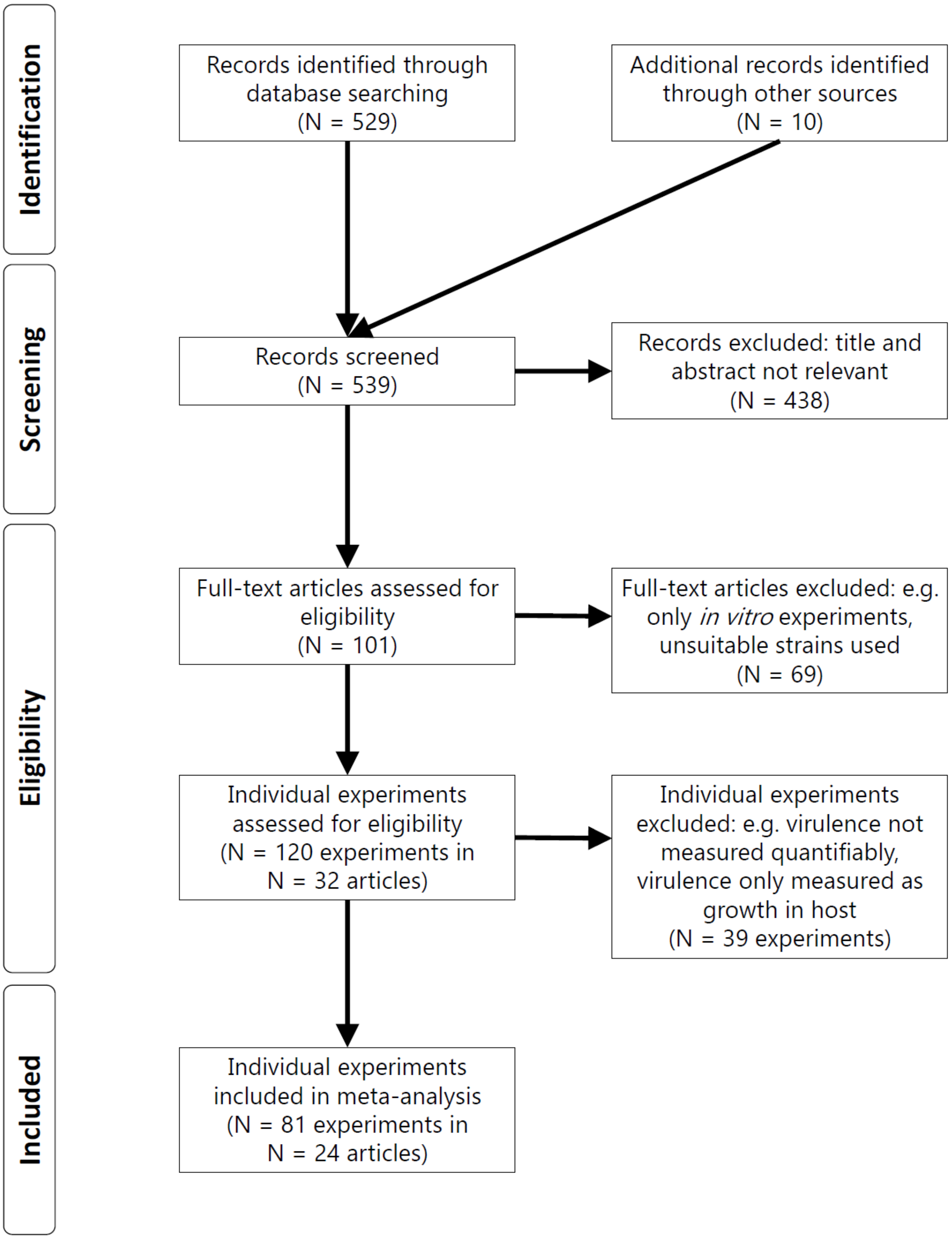

Fig. S1 Flow diagram (PRISMA format) of the screening and selection process for studies investigating the association between pyoverdine production and virulence in *P. aeruginosa*.
