# Supplementary material for: Do Bacterial “Virulence Factors” Always Increase Virulence? A Meta-Analysis of Pyoverdine Production in Pseudomonas aeruginosa As a Test Case
Source: Front Microbiol. 2016 Dec 12;7:1952. doi: 10.3389/fmicb.2016.01952 (PMC5149528; doi:10.3389/fmicb.2016.01952)
Supplement: Supplementary file 6 [file Image2.PDF]

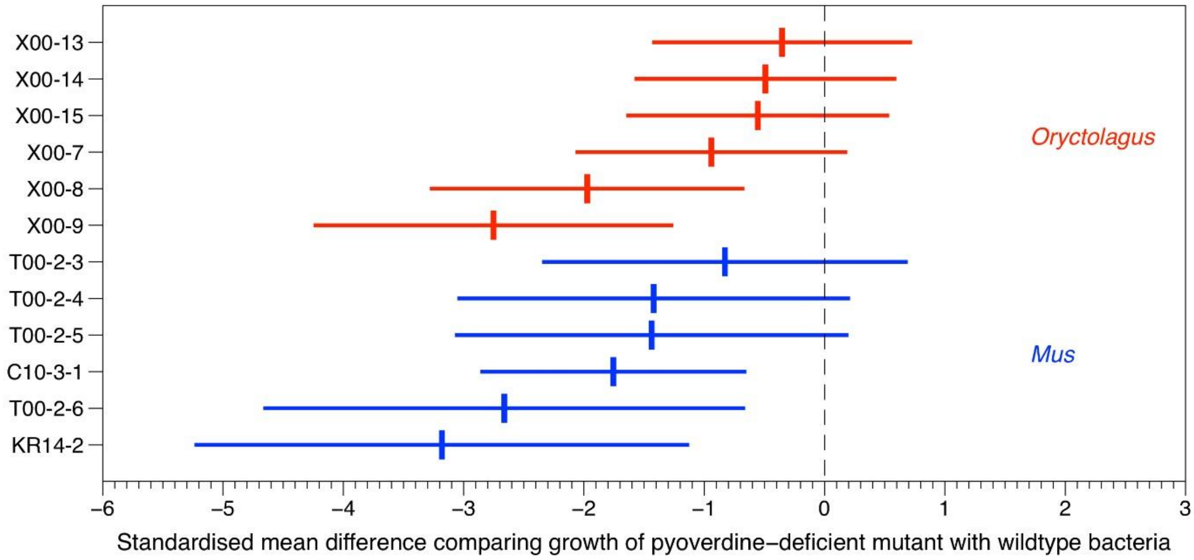

Fig. S2 Forest plot depicting the variation in effect size across experiments on the effect of pyoverdine on the growth of *P. aeruginosa* in mammalian hosts. Effect sizes are given as standardized mean difference ± 95% confidence interval and are grouped by host genus. Negative and positive effect sizes indicate lower and higher *in vivo* growth of the pyoverdine-deficient mutant relative to the wildtype, respectively. IDs of the individual experiments are listed on the Y-axis (for details, see Table S4 in the supplemental material).
